# Management of pain in cancer patients— lessons from practices during the COVID-19: a qualitative study of cancer care providers’ perspectives

**DOI:** 10.1186/s12913-024-10710-z

**Published:** 2024-02-22

**Authors:** Georgina Cornall, Emma Zhao, Tim Luckett, Ertugrul Erciyas, David Monck, Paul Glare, Andy Wang, Yi-Ching Lee

**Affiliations:** 1 Sydney Medical School, Faculty of Medicine & Health, The University of Sydney, Sydney, Australia; 2 Sydney Nursing School, Faculty of Medicine & Health, The University of Sydney, Sydney, Australia; 3 Department of Anaesthetics and Pain Management Centre, Royal Prince Alfred Hospital, Sydney, Australia; 4 Department of Anaesthesia and Pain Service, Chris O’Brien Lifehouse, Sydney, Australia; 5 IMPACCT (Improving Palliative, Aged and Chronic Care through Clinical Research and Translation), Faculty of Health, University of Technology Sydney (UTS), Sydney, Australia

**Keywords:** Pandemic, Delivery of health care, Health services: needs and demands, Disease management

## Abstract

**Background:**

The ongoing COVID-19 pandemic has impacted health systems globally and affected managing many chronic conditions, including cancer. This study aimed to explore the perceptions of multi-disciplinary cancer care providers on how cancer pain management was affected by the COVID-19 pandemic.

**Methods:**

Participants were eligible if they were cancer care providers of any specialty and discipline from two tertiary hospitals in Australia. Data were collected using semi-structured interviews to explore cancer care providers’ perspectives on cancer pain management within COVID-19. Thematic analysis of interview transcripts used an integrated approach that started with inductive coding before coding deductively against a behaviour framework called the COM-B Model, which proposes that ‘capability’, ‘motivation’ and ‘opportunity’ are requisites for any behaviour.

**Results:**

Twenty-three providers participated. Five themes were developed and interpreted from the analysis of data, namely: “Telehealth enables remote access to cancer pain management but also created a digital divide”, “Access to cancer pain management in the community is compromised due to the pandemic”, “COVID-19 negatively impacts hospital resource allocation”, “Patients were required to trade off cancer pain management against other health priorities” and “Hospital restrictions result in decreased social and psychological support for patients with cancer pain”.

**Conclusions:**

The landscape of cancer pain management in the Australian health system underwent substantial shifts during the COVID-19 pandemic, with lasting impacts. Cancer care providers perceived the pandemic to have significant adverse effects on pain management across multiple levels, with repercussions for patients experiencing cancer-related pain. A more adaptive health system model needs to be established in the future to accommodate vulnerable cancer patients.

**Supplementary Information:**

The online version contains supplementary material available at 10.1186/s12913-024-10710-z.

## Background

Pain is one of the most common symptoms present at cancer diagnosis, and its prevalence increases over the course of treatment and as the disease progresses [1]. Of patients with cancer, over half will suffer pain and one-third with moderate to severe intensity [2]. Pain in people with cancer is deleterious to quality of life due to impacts on physical, psychological, and social functioning [1, 2]. Effectively managing cancer pain requires comprehensive assessment and a multi-disciplinary approach to care [1].

In December 2019, the first case of SARS-CoV2 (COVID-19) was reported in Wuhan, China. Since then, COVID-19 has impacted health systems globally, including in Australia [3]. Whilst deaths from COVID-19 in Australia have been relatively low compared to many other countries, the health system was substantially impacted by public health measures introduced to slow down the spread of the disease [3]. These included a wide-scale extension of reimbursement for telehealth to replace face-to-face consultations with primary and secondary care providers, [4] redeployment of staff, reductions in screening programs, restrictions on elective surgeries and suspension of some treatments [3] and visitor restrictions for hospital inpatients [5]. Cancer patients have an elevated risk of mortality or developing severe symptoms from COVID-19 [5]. Public policy and patients’ fear of COVID-19 have combined to reduce face-to-face presentations to cancer services, hospitals [6, 7] and general practice, [8] with a rise in demand for telehealth consultations and community care [7, 8]. Physical distancing orders have increased patients’ social disconnectedness, increasing anxiety for cancer patients, carers, and families [5].

Whilst the above summary outlines the impacts of the COVID-19 pandemic on cancer care in general, its impacts on cancer pain management, more specifically, have not been investigated [8]. The current study aimed to explore the perceptions of multi-disciplinary cancer care providers on how cancer pain management was affected by the COVID-19 pandemic. By exploring the effects of the pandemic on cancer pain management, this study aimed to determine potential avenues of support for patients with cancer with a view to examine the impact on their pain management and enhance continuity of care.

## Methods

### Aim & Design

The study took a qualitative approach to allow an in-depth exploration of the experiences and attitudes of providers regarding the influence of COVID-19 on cancer pain management. We used an integrated approach that combined inductive with deductive components to ensure that results built on previous understanding whilst remaining open to unexpected insights [9]. Reporting of the study adheres to the consolidated criteria for reporting qualitative research (COREQ) [10].

Ethics approval was obtained from the Sydney Local Health District Human Research Ethics Committee, approval number: X21–0312 & 2021/ETH11313. All participants gave written informed consent before participating. All research procedures were in accordance with the Declaration of Helsinki.

### Setting and participants

Eligible participants were cancer care providers at two tertiary teaching hospitals directly providing in-patient and/or outpatient care for patients with cancer-related pain. Participants were recruited using email invitations through clinical departments. Permission was sought from clinical departments involved in cancer pain management to send out invitations regarding the study on behalf of the research team. Those interested in participating were further contacted by research staff individually to explain the study further. We included participants from any specialty and discipline to explore a fuller range of perspectives. Snowball sampling was implemented by encouraging participants to invite colleagues to participate [11].

### Data collection

Data were collected via semi-structured interviews conducted face-to-face and over Zoom video-conferencing. Interviews were conducted by two medical students, one female (GC) and one male (EE), between February and March 2022. Neither interviewer had prior experience in qualitative research but received guidance from an academic social scientist with substantial experience (TL). No prior relationships existed between participants and interviewers. An interview guide was developed by the research staff and aimed to allow the elicitation of perspectives and experiences of participants (Supplementary Table 1). The guide was constructed around open-ended questions with probes to explore further questions. Each interview was audiotaped and lasted between 7 and 45 minutes. No interviews were repeated. Handwritten field notes were taken during interviews to supplement transcripts and summarise key discussion points.

### Data analysis

Transcriptions were managed in the program ‘NVIVO’ (Release 1.6.1). Analysis used an integrated approach specifically designed to explore how cancer pain management was affected by the COVID-19 pandemic. Each transcription was read carefully and compared with audio recordings to ensure accuracy, then coded inductively and descriptively. Two researchers (EE and GC) completed the coding of all transcripts to develop an initial codebook. Following this, GC performed deductive coding to code against categories according to an established framework for understanding behaviour called the COM-B Model, which proposes that ‘capability’, ‘motivation’ and ‘opportunity’ are pre-requisites for any behaviour [12, 13]. The COM-B model is the most widely used theoretical framework for analysing behaviour in health, both for patients and health care professionals, including cancer pain. It is based on a systematic review and synthesis of 19 behaviour change frameworks and has the potential to identify system- and service- as well as clinician- and patient-level interventions that will be useful for improving outcomes [12]. Themes were derived from data analysis and discussed and agreed on by two researchers (GC and EE) to enrich interpretation and guard against bias from one person’s perspective. Subthemes within the core themes were summarised alongside illustrative quotations from participants identified with a participant number (Supplementary Table 2) in Table 2.

## Results

Twenty-three participants were interviewed. They were predominantly medical officers (*n* = 12) and female (*n* = 14) and had a median of 15 years of clinical experience (inter-quartile range = 14, Table 1).

**Table 1:** Characteristics of interviewed participants (*n* = 23)

Characteristics	*N* (%)
Female	14 (61)
Clinical role	
Medical	12 (52)
Nursing	9 (39)
Allied health	2 (9)
*Pharmacist*	*1 (4)*
*Music therapist*	*1 (4)*
Medical specialty	
Anaesthetist	4 (17)
Oncologist (medical and radiology)	2 (9)
Palliative care physician	2 (9)
Intensivist	1 (4)
Pain management physician	1 (4)
I preferred not to say	1 (4)
Cancer patients seen per week	
> 40	6 (26)
31–40	2 (9)
21–30	3 (13)
10–20	8 (35)
< 10	2 (9)
Not recorded	2 (9)

Five themes were developed and interpreted from the analysis of data collected: “Telehealth enables remote access to cancer pain management but also created a digital divide”, “Access to cancer pain management in the community is compromised due to the pandemic”, “COVID-19 negatively impacts hospital resource allocation”, “Patients were required to trade off cancer pain management against other health priorities” and “Hospital restrictions result in decreased social support for patients with cancer pain”. Subthemes within each theme and illustrative quotations are categorised according to the COM-B model (Table 2) [12].

**Table 2:** Thematic framework: Themes, COM-B categories and subthemes supported by example quotations

Themes	COM-B category	Subthemes	Example quotations
**Telehealth enables remote access to cancer pain management but also creates a digital divide**	Physical opportunity	Telehealth enables remote access to healthcare	*“…the telehealth helps. I mean, it’s almost better in some ways, the patient doesn’t have to go all the way to their GP [general practitioner] or to the surgeon.” (C12, Clinical Nurse Consultant)* *“…it is a very useful tool when people can’t come in, and it’s good for country patients because before they would have to come down to Sydney.” (C23, Clinical Nurse Consultant)*
		Telehealth limits the ability of clinicians to assess and examine patients	*“Because you can’t see them in person and it’s hard to assess how well someone is through video conference.” (C08, oncology Junior Medical Officer)*
	Psychological capability	Difficulty using telehealth to assess pain experienced by patients	*“…[I] don’t have the same rapport or the same therapeutic relationship. I think there’s a lot you can get from just seeing a patient within just a few seconds. You can see how well they’re doing, how their pain is.” (C15, palliative care registrar)*
		Communication over telehealth is limited	*“I think it can be hard for patients to explain their [pain] experiences over telehealth” (C09, Junior Medical Officer)* *“It’s much harder to appear genuine or empathic or invested in listening when you’re online or even on a telephone. It’s just another voice on the end of the line.” (C14, anaesthetic specialist)* *“A lot of the telehealth that I’ve provided has been just the old-fashioned telephone. So, there’s been no video calling, it’s just been a conversation over a telephone, and that can be improved.” (C14, anaesthetic specialist)*
		Challenges transferring clinical skills to telehealth	*“So, the biggest change has been getting better at assessing through a non-face-to-face method.” (C13, anaesthetic registrar)*
		Lack of patient skills using telehealth technology	*“…when you’re dealing with an older demographic that perhaps… don’t understand how to do things a bit more virtually and use technology.” (C07, music therapist)*
**Access to cancer pain management in the community is compromised due to the pandemic**	Physical opportunity	Limited access to face-to-face appointments with general practitioners	*“…medical centres and GPs [general practitioners] have been closed, or only doing remote stuff.” (C19, anaesthetic specialist)*
		Limited access to community health services impacts timely management	*“…a lot of clinical nurse consultants were taken out of their regular roles, which is outreach to patients in the community and made to work in different departments …so those patients that they were normally following up with, cancer patients or other patients, have missed that follow up…” (C21, Nursing Unit Manager)*
**COVID-19 negatively impacts hospital resource allocation**	Physical opportunity	Impact of COVID-19 restrictions on elective surgery for cancer pain	*“So, what has happened is that elective lists have been cancelled. And amongst that interventional pain lists are part of the elective process. So, something like cancer pain management, I think should be prioritised because of the significant impact that they can have on their care.” (C13, anaesthetic registrar)*
		Prioritisation of beds for COVID-19 management	*“Beds have been short because even in the cancer hospital there’s been an overflow of general patients from the main hospital that required beds for management and treatment of their COVID.” (C14, anaesthetic specialist)*
		Redeployment of hospital staff	*“There were a lot of staff changes lately due to redeployment.” (C17, pain management specialist)*
**Patients were required to trade off cancer pain management against other health priorities**	Reflective motivation		*“I think it was just mainly fear that they’re going to come in and they’re going to catch COVID off either a staff member or another patient.” (C04, Enrolled Nurse)* *“…there’s been a lot of COVID fear… And that has actually stopped people from going to emergency with pain.” (C12, Clinical Nurse Consultant)*
**Hospital restrictions result in decreased social and psychological support for patients with cancer pain**	Social opportunity		*“I think that, from the psychological side, all the patients just get more and more isolated…they’re more miserable. They have less access to family.” (C18, intensivist registrar)* *“…because of personal protective equipment and isolation requirements, families might not be there, so they’re psychologically more isolated.” (C10, Junior Medical Officer)*

### Telehealth enables remote access to cancer pain management but also creates a digital divide

#### Physical opportunity

Some participants perceived telehealth helped patients avoid travelling to access care for cancer-related pain. This was perceived to be particularly beneficial for patients from rural communities. Furthermore, one participant noted that *“it [telehealth] means that the patients are in the comfort of their own home” (C06, Clinical Nurse Specialist)*.

On the other hand, telehealth was perceived to be suboptimal in ways that negatively impacted pain management, including a reduced ability to assess and examine patients, including the capacity to read patient expressions. One participant found that *“the GPs [General Practitioners] are reluctant, as they are ‘telehealthing’ to chart any kind of opioids [for cancer pain] because they’re not seeing them in person.” (C12, Clinical Nurse Consultant).*

#### Psychological capability

Some participants identified that the use of telehealth reduced their ability to build rapport with patients, especially when conducted by telephone rather than video-conference. In addition, most participants reported being less able to recognize patient cues and expressions contributing to their assessment of cancer pain and general health. One participant mentioned that *“…the fact that they’ve seen no one face to face and no one actually identifies how much pain [cancer patients] are in, unless they ask the question.” (C01, anaesthetic specialist)* Some participants highlighted that, unless directly questioned, some patients might not report being in pain or may under-report their cancer pain, leading to it going unidentified. One of the oncology specialists stated, “*I truly think that when it comes to managing patients’ symptoms, and I think pain, it should be done by video call because you need to see the patient’s expression. They might be downplaying a lot of things when they’re in pain.” (C22).*

Some participants also perceived the clinician-patient communication and therapeutic relationship as negatively impacted by telehealth, both in terms of engaging with patients empathetically and enabling patients to communicate their experience fully. Participants observed that the psychosocial implications of pain required a level of human interaction only possible through face-to-face consultations. One participant explained that *“because cancer pain is not only biological and diagnostic pain, it also has other implications, psychosocial implications. So those people you need to have an interaction like human to human, face to face interaction for giving good quality comfort care, which I don’t think telehealth can completely replace.” (C10, Junior Medical Officer)*.

Furthermore, some participants observed that telehealth was impeded by a lack of skills both on their part and that of patients. They perceived it difficult to transfer clinical skills to the telehealth medium and found older patients struggled with technology, especially videoconferencing. For instance, *“…patients have difficulty getting on to the video telehealth consult. The other thing is that elderly patients are not particularly IT savvy, so they can have difficulty as well.” (C22, oncology specialist).*

### Changes in accessing cancer pain management at a community level

#### Physical opportunity

A small proportion of participants noted that COVID-19 had seen a reduction in face-to-face appointments offered by general practitioners and a reduction in community healthcare services thereby negatively impacting the care of patients with cancer pain. One participant also suggested that *“…there definitely needs to be more resources in the community. I think the focus really should be allowing access to pain management strategies, specialists, palliative care, pain management at home.” (C15, palliative care registrar).*

### Impacts of COVID-19 on hospital resource allocation

#### Physical opportunity

Several participants noted government COVID-19 restrictions on elective surgeries in hospitals significantly impact the management of cancer pain because these included interventional procedures, despite these being viewed as a priority. One *pain management specialist said, “…so, they [hospital] stopped elective surgery…. We have managed to do some cancer pain interventions on the emergency list…So it’s still feasible to do, but it’s just not very efficient.” (C17).*

Additionally, most participants identified reallocating hospital beds for COVID-19 patients and staff shortages as key issues, with hospital staff being redeployed to vaccination clinics, health hotels, other departments, and wards. *“A lot of cancer nurses would have been redeployed to RPA virtual, contact tracing, vaccination clinics, and intensive care and special health accommodation.” (C20, Clinical Nurse Consultant).*

### Perceptions of COVID-19 and balancing health priorities

#### Reflective motivation

Most participants observed that many patients with cancer pain were apprehensive about presenting to the hospital due to fear of exposure to COVID-19 from staff or other patients. In the context of a cancer pain exacerbation, this was described by one participant as contributing to prolonged suffering from pain. *“People having pain crises or exacerbations of their pain couldn’t come in as readily…. Patients are often reluctant to present even when they were in pain, so you’d find they were suffering for many days or weeks because of the fear.” (C15, palliative care registrar).*

### Hospital restrictions and implications for social support for patients with cancer pain

#### Social opportunity

Several participants described the important role that visits from family and friends played in providing social support to cancer inpatients. Participants noted that restrictions on hospital visitors implemented during COVID-19 had psychological implications for cancer patients with pain. One palliative care registrar mentioned that *“there’s a lot of psychological factors in pain management…not being allowed to have family members present during the pandemic has really impacted the experience of pain. I think people have suffered more.” (C15).*

## Discussion

This study explores cancer care providers’ perspectives on the impact of COVID-19 on managing pain in patients with cancer in Australian hospitals. Participants perceived the pandemic to have significant adverse effects on pain management across multiple levels, with repercussions for patients experiencing cancer-related pain. The findings in our study are consistent with current literature regarding the impact of the pandemic on pain management. In addition to these findings, we identified unique challenges faced by patients with cancer, especially those in the active treatment phase and those with a terminal diagnosis.

The widespread uptake of telehealth was perceived to improve access to cancer pain management and allow access to healthcare from a comfortable environment. In the past, tele-supervision, tele-trial and tele-chemotherapy have shown promising results in delivering care in regional and low-resource health settings [14, 15]. In other research, telehealth has been found to reduce the necessity of patients to travel to appointments, thereby minimising transmission of COVID-19 during the pandemic [4, 6]. Additionally, providing care in a familiar environment using telehealth creates an avenue for tailored and patient-orientated care [16].

Telehealth has improved access and shown evidence of discrepancies between patients and healthcare providers in acceptance of explaining physical examinations by this method in different healthcare settings even before the COVID-19 pandemic [14, 15, 17]. Telehealth is particularly useful for patients who are known to the pain management team and require none or minimal physical examinations. It is more beneficial and convenient for both patients and healthcare providers to discuss chronic pain conditions and make treatment plans through telehealth [18, 19]. Moreover, it has reported no differences in service delivery and standard of care whether patients had telehealth or in-person physical examinations [18, 20]. However, certain aspects of face-to-face consultations were perceived to be inaccessible through telehealth, especially for those patients who require pain procedures or some specific examination [19]. Identifying clinical problems may be limited by the inability to physically examine patients when utilising telehealth, particularly telephone-based telehealth [21]. Furthermore, a lack of physical examination of oncology patients may result in patient perceptions of reduced quality of care and fears of inaccurate assessment of wellbeing [22]. Whereas, previous studies have proposed that videoconferencing be used in conjunction with consultations conducted via telephone to allow clinicians to recognise patient expressions and cues [23]. Clinical guidelines in using a mixed model of videoconferencing, telephone and in-person care should be considered in future practice to improve cancer pain management even beyond the context of the pandemic.

The future role of telehealth in pain management as the world enters a new phase of the pandemic requires careful consideration. As the government in Australia moves towards reducing the utilization of telehealth in pain management (with the removal of Medicare Benefits Schedule item numbers for telephone consultations for pain management), no exemption or adaptation was made to accommodate the vulnerable cancer patient population. While it is reasonable to encourage improvements in functional levels and in-person attendance to consultations for patients with chronic pain, [24] the needs of patients with cancer are different. Time toxicity [25]-spending unnecessary time in the waiting room, travelling to attend consultations, dealing with limited life expectancy, and potentially risky exposure to infectious elements in the community while immunosuppressed, are visible examples of negative impacts. Additionally, cancer patients’ culturally and linguistically diverse (CALD) backgrounds need a collaborative approach, such as virtual translators, to maximise the support and health benefits [26].

Participants in the current study also perceived technological literacy contributed to the digital divide that emerged in cancer pain management during the pandemic. Widespread use of telehealth may lead to reduced outcomes for certain groups, such as patients from culturally and linguistically diverse backgrounds and those with lower health and technological literacy [4]. These issues are especially important to consider in the context of opioid prescribing for cancer pain. Racial disparities in opioid prescribing for chronic pain have been well-researched, and clinicians’ patterns of prescribing may be influenced by their own biases towards minority patients [27]. In this study, it was perceived that general practitioners were hesitant to prescribe opioids for cancer pain over telehealth. It is therefore important to consider if hesitancy by clinicians to prescribe opioids during telehealth consultations and existing racial disparities in opioid prescribing could result in a double disadvantage for specific patient groups. Further research into the interplay of such disparities and the widespread use of telehealth in cancer pain management and indeed, more widely chronic pain, requires further research as telehealth continues to be widely utilised beyond the pandemic era.

It is well established that cancer pain does not exist in isolation but rather is influenced by psychological, social, and physical factors that contribute to a patient’s experience of pain [28]. Participants in this study provide new insights into the impact of the COVID-19 pandemic on cancer patients’ experience of cancer pain. Hospital visitor restrictions were perceived to result in a loss of social support for patients with cancer, contributing negatively to pain experienced, especially in the context of patients with advanced disease. Restrictions, while beneficial for reducing the transmission of COVID-19, were perceived to heighten patient distress and limit the psychological benefits normally available through social support [23]. Similarly, healthcare measures introduced during the pandemic have been recognised to have significant ramifications on chronic pain management, with patients unable to access certain treatments that may have been considered ‘non-essential’ [29]. Social isolation, increased anxiety, and COVID-19 restrictions have been found to influence chronic pain experienced by patients negatively, further highlighting the link between pain and biopsychosocial factors [29].

The removal of a patient’s support system and the added disadvantages of telehealth limiting physicians’ ability to physically provide support in the form of touch and non-verbal communication of empathy, negatively impact the psychosocial well-being of cancer patients [4, 23]. Providers in this study perceived that in consideration of the psychosocial factors that both influence and arise from cancer pain, telehealth cannot fully replicate the human interaction that is required to provide “good quality comfort care” (C10) to patients with cancer pain. Given these findings, there may be a benefit in providing cancer pain management through a hybrid of both virtual and in-person care. For example, Royal Prince Alfred (RPA) Virtual Hospital was developed to provide remote healthcare and observation of patients with COVID-19 who were stable [30]. This system allowed monitoring of vital signs, video-based consults with nursing staff and access to 24/7 virtual care [30]. A similar system developed by the Mayo Clinic in the USA, Advanced Care at Home, utilised virtual hospital care but also in-person home visits to allow delivery of medication, collection of laboratory samples and physical assessments [31]. Implementing a similar model of care for cancer pain management that combines virtual and in-person management during the pandemic may provide a novel solution for increasing social support and improved access through providing home-based cancer pain management while allowing physicians to support and assess patients adequately physically.

Patients’ fear of COVID-19 was an added complexity in providing care for patients with cancer pain due to the psychological and social pressures that arose during the pandemic. The pandemic placed pressure upon cancer patients to balance different health priorities, management of their cancer and minimising the risk of COVID-19 exposure [32]. Cancer patients delayed or cancelled consultations and had an increased threshold for attending hospital and delayed treatment in response to the risk of contracting COVID-19 [3, 6]. Importantly, in this study, providers reported that patients were apprehensive about seeking medical care for cancer pain exacerbations due to anxiety about being exposed to COVID-19, resulting in delays in treatment. The development of a model of care that allows access to improved virtual care and in-person cancer pain treatment at home could reduce the need for patients to attend healthcare settings where risks of exposure to transmissible diseases are heightened.

Within the community, it was identified by providers that there were reduced face-to-face consultations for patients with cancer pain across community health services and general practice clinics. There is limited literature to support this, however, the full impacts of the shift to telehealth and reduced face-to-face consultations on patient access to care provided by general practitioners during the pandemic are still emerging [8]. Providers in this study expressed a need for more resources in the community for cancer pain management and improved access to cancer pain services for patients from home.

At a hospital level, government COVID-19 restrictions on elective procedures forced some cancer pain procedures, including interventional procedures, to be performed on an emergency list. However, as noted by participants, those pain intervention procedures were completed by delaying other emergencies. Resources required for surgery, such as beds and staff, were primarily directed towards the management of COVID-19 patients [33]. Acute care became a focus during the pandemic, as reflected by these changes, and interventional procedures usually offered for chronic pain became increasingly difficult to perform, given restrictions on elective procedures and redistribution of resources during the pandemic [29, 34]. COVID-19 also impacted resources more generally across hospital settings, with participants in this study describing how resources such as beds were prioritised to manage COVID-19 patients, further impacting the management of cancer pain. Treatments for chronic pain, such as multidisciplinary management strategies and physical therapy, were identified in previous studies to become increasingly difficult to access, resulting in treatment delays for chronic pain patients [29]. Additionally, aligning with previous literature, [35] hospital staff were redeployed to assist in COVID-19 healthcare services. Providers noted that this reduced staff available to manage cancer-related pain.

## Conclusion

In conclusion, the COVID-19 pandemic has significant adverse effects on cancer pain management across multiple levels. Telehealth can allow cancer pain patients to access pain management services and health care resources remotely, even though social and psychological support may be issued due to hospital restrictions. Clinical guidelines and a more adaptive health system model need to be established in the future to accommodate the different needs of patients with cancer-related pain.

### Strengths and limitations

This study highlights the importance of considering the psychosocial aspects of cancer pain when providing treatment. Implementing a novel model of care that combines video-based virtual and in-person management of cancer pain could enable remote treatment in a comfortable, supportive environment. This can also ensure the ability of physicians to physically assess patients’ cancer pain and deliver medication and treatment. This model could also have the advantage of minimising patient exposure to COVID-19 and other ongoing infectious disease threats, such as influenza, in healthcare settings. In turn, it may potentially minimise delays in treatment for cancer pain caused by fear of infection, which remain prominent for many people at heightened risk of poor outcomes. Increasing access to cancer pain management in the community could also reduce the impact of COVID-19 and any future pandemics on hospital resources and ensure emergent treatment and procedures remain readily available to patients with cancer pain.

This study has several limitations. Firstly, this study did not include patient perspectives. Secondly, considering the limited sample size of participants recruited from two quaternary hospitals in NSW, Australia, the perspectives reported may not be fully generalisable to larger populations. Thirdly, clinical disciplines were overrepresented in the study, including medical and nursing disciplines, compared with allied health professionals. Lastly, due to the staff availability and invitation responses, only two oncologists were recruited in this study.

Despite the limitations, there are several strengths to this study. The recruitment and sampling methods yielded a wide range of perspectives presented in the study with a balance of different healthcare professions, years of clinical experience and medical specialties.

### Supplementary Information

**Supplementary Material 1.**

## Data Availability

The data supporting this study cannot be publicly shared for ethical reasons but may be shared upon reasonable request made to the corresponding author.

## Abbreviations

COM-B: Capabilities, opportunities, motivation - behaviour
COVID-19: SARS-CoV2
COREQ: Consolidated criteria for reporting qualitative studies
RPA: Royal prince alfred
