# Supplementary material for: Management of pain in cancer patients— lessons from practices during the COVID-19: a qualitative study of cancer care providers’ perspectives
Source: BMC Health Serv Res. 2024 Feb 22;24:232. doi: 10.1186/s12913-024-10710-z (PMC10885360; doi:10.1186/s12913-024-10710-z)
Supplement: Supplementary file 1 — Supplementary Material 1. [file 12913_2024_10710_MOESM1_ESM.pdf]

Supplementary Table 1: Interview Guide for Semi-structured Interviews

Construct	Key Questions	Prompts
Management of patients with cancer pain during the Covid-19 pandemic	1. What are major difficulties or changes for you during the pandemic in the management of patients with cancer pain?	e.g., availability of staff; workload; changes of work strategies (zoom/phone call follow up), access to resources, elective surgery restrictions, reduced beds in palliative care
	2. What are the major issues in the management of patients with cancer pain because of the pandemic?	e.g., timely treatment, hospital resources, lack of physical examination due to telehealth consultation, fear on behalf of patients to come to the hospital, restrictions on hospital visitors
	3. What do you think can potentially be improved?	e.g., Access to health care resources:<br>- hospital level (obtaining a bed for admission)<br>- community level (availability of community palliative care nurse or Allied health)

Supplementary Table 2: **Participant Identification Key**

Participant ID Number	Clinician Type
C01	Specialist, Anaesthetist
C02	Registered Nurse
C03	Clinical Nurse Specialist
C04	Enrolled Nurse
C05	Registered Nurse
C06	Clinical Nurse Specialist
C07	Music therapist
C08	Junior Medical Officer, Oncologist
C09	Junior Medical Officer
C10	Junior Medical Officer
C11	Pain pharmacist
C12	Clinical Nurse Consultant
C13	Registrar, Anaesthetist
C14	Specialist, Anaesthetist
C15	Registrar, Palliative care
C16	Specialist, Palliative care
C17	Specialist, Pain management
C18	Registrar, Intensivist
C19	Specialist, Anaesthetist
C20	Clinical Nurse Consultant
C21	Nursing Unit Manager
C22	Specialist, Oncologist
C23	Clinical Nurse Consultant

(Further information for each participant identified by a participant identification number that was assigned to example quotations from participants presented in Table 2)
